# Wastewater Surveillance to Confirm Differences in Influenza A Infection between Michigan, USA, and Ontario, Canada, September 2022–March 2023

**DOI:** 10.3201/eid3008.240225

**Published:** 2024-08

**Authors:** Ryland Corchis-Scott, Mackenzie Beach, Qiudi Geng, Ana Podadera, Owen Corchis-Scott, John Norton, Andrea Busch, Russell A. Faust, Stacey McFarlane, Scott Withington, Bridget Irwin, Mehdi Aloosh, Kenneth K.S. Ng, R. Michael McKay

**Affiliations:** University of Windsor, Windsor, Ontario, Canada (R. Corchis-Scott, M. Beach, Q. Geng, A. Podadera, O. Corchis-Scott, K.K.S. Ng, R.M. McKay);; Great Lakes Water Authority, Detroit, Michigan, USA (J. Norton, A. Busch);; Oakland County Health Division, Oakland, Michigan, USA (R.A. Faust);; Macomb County Health Department, Macomb, Michigan, USA (S. McFarlane);; Detroit Health Department, Detroit (S. Withington);; Windsor-Essex County Health Unit, Windsor (B. Irwin, M. Aloosh);; McMaster University, Hamilton, Ontario (M. Aloosh)

**Keywords:** influenza, influenza A virus, epidemiology, vaccination, prevalence, viruses, respiratory infections, wastewater surveillance, Michigan, Ontario, Canada, United States

## Abstract

Wastewater surveillance is an effective way to track the prevalence of infectious agents within a community and, potentially, the spread of pathogens between jurisdictions. We conducted a retrospective wastewater surveillance study of the 2022–23 influenza season in 2 communities, Detroit, Michigan, USA, and Windsor-Essex, Ontario, Canada, that form North America’s largest cross-border conurbation. We observed a positive relationship between influenza-related hospitalizations and the influenza A virus (IAV) wastewater signal in Windsor-Essex (ρ = 0.785; p<0.001) and an association between influenza-related hospitalizations in Michigan and the IAV wastewater signal for Detroit (ρ = 0.769; p<0.001). Time-lagged cross correlation and qualitative examination of wastewater signal in the monitored sewersheds showed the peak of the IAV season in Detroit was delayed behind Windsor-Essex by 3 weeks. Wastewater surveillance for IAV reflects regional differences in infection dynamics which may be influenced by many factors, including the timing of vaccine administration between jurisdictions.

The SARS-CoV-2 pandemic reasserted the importance of epidemic preparedness and surveillance systems for infectious diseases (*1*). Informed responses to public health challenges require that data be available to decision-makers in a timely manner for early interventions (*1*). However, traditional clinical based measures of disease incidence have limited use in providing early warnings. Relying on influenza-like illness data is problematic because of difficulty distinguishing between infections ascribed to influenza A virus (IAV), influenza B virus, SARS-CoV-2, or respiratory syncytial virus (*2*). Virologic surveillance enables respiratory illness to be classified on the basis of etiologic agent. However, results are often slow, and interpretation must account for factors such as test-seeking behavior, accessibility of healthcare services, severity of infection, diagnostic practices of healthcare providers, and hospital protocols. In addition, laboratory capacity may be exceeded, and testing is expensive (*3*,*4*). 

Wastewater surveillance (WS) is shown to be a practical approach for disease surveillance at various spatial scales, offering effectiveness and economic advantage (*5*,*6*). WS for SARS-CoV-2 relies on quantifying viral RNA shed in feces and has substantially increased in use since its implementation to track infections during the COVID-19 pandemic. Studies have found the concentration of SARS-CoV-2 RNA in municipal sewage covaries with the levels of disease circulating within the community served and can predict trends in clinical cases and hospitalizations (*7*,*8*). In addition, WS has the potential to be rapid; sample processing, measurement, analysis, and dissemination of results took <6 hours in Windsor-Essex, Ontario, Canada. Calls have been made to expand the scope of WS to include monitoring of IAV and other endemic respiratory pathogens that are underreported (*9*,*10*). Similar to SARS-CoV-2, IAV can be shed in feces (*11*), and recent studies have used WS to track IAV (*12*–*14*). However, more work needs to be done to validate WS compared with traditional surveillance metrics. WS can be useful in measuring regional differences in infection dynamics and understanding how IAV and other pathogens spread across jurisdictional boundaries.

The Detroit-Windsor metropolitan area, encompassing the cities of Detroit, Michigan USA, and Windsor-Essex, Ontario, Canada, is North America’s largest transborder conurbation and is the busiest cross-border region for trade between the United States and Canada, handling 42% of commercial traffic between Ontario and Michigan and accounting for ≈25% of total daily commercial traffic between the United States and Canada (*15*). The region is a major entry point for visitors, including >5,000 commuters from Windsor-Essex who cross the border daily for work (*15*,*16*). Detroit and Windsor-Essex represent government structures and public health jurisdictions that adopted fundamentally different vaccination and mitigation strategies during the COVID-19 pandemic. The cessation of COVID-19 pandemic mitigation strategies, such as masking and social distancing, affected the circulation of respiratory pathogens other than SARS-CoV-2, such as IAV. The end of those mitigation strategies resulted in a delayed start to the 2021–22 influenza season in Windsor-Essex, which coincided with the removal of mask mandates in Ontario in March 2022, and may have had a role in unusual patterns of influenza incidence during the 2022–23 influenza season in Canada (*17*,*18*). Similarly, the 2021–22 influenza season in Michigan was mild, with an increase in influenza activity observed in November, followed by a decline in January 2022 and a subsequent rise in activity in March 2022. Trends in Michigan were similar to national trends; levels of influenza activity remained elevated through mid-June 2022 (*19*,*20*). Those unusual patterns of influenza prompted this retrospective investigation comparing the incidence of influenza in Windsor-Essex with the incidence of influenza in Detroit. Our goal is to understand how jurisdictional differences in pandemic mitigation strategies and public health policy influenced the timing of influenza seasons. 

The initial investigation into influenza hospitalization data for Windsor-Essex and Michigan through a visual inspection of the data showed a delay in the onset and peak of the 2022–23 influenza season in Michigan compared with Windsor-Essex. Because influenza incidence data (including hospitalizations) specific to Detroit are not publicly available and influenza is an underreported disease, the trend observed through examination of clinical data may not be sufficient to claim a delayed onset in the influenza season. Because WS is based on the aggregated waste of an entire community, it is anonymous and reflects population level trends that could produce a more sensitive and non-biased measure of influenza incidence to confirm trends in clinical data. Analysis of WS data, coupled with traditional measures of disease incidence, will enable a more complete understanding of how differences in public health approaches in a divided, yet contiguous, metropolitan area influenced the trajectory of the influenza season after COVID-19 mitigation policy removal.

## Methods

### Sample Collection

During September 1, 2022–March 31, 2023, we collected composite (24-h) wastewater samples 3–5 days/week from 2 different wastewater treatment plants that serve a resident population of ≈270,000 persons, ≈50% of the regional population, located in Windsor-Essex. In parallel, we collected composite samples 1 day/week from the 3 interceptors terminating at the Water Resource Recovery Facility (WRRF) operated by the Great Lakes Water Authority (GLWA), located in Detroit (*21*) (Appendix). The WRRF serves the entire city of Detroit and treats the waste of ≈3 million people, 88% of the residents in the greater Detroit metropolitan area and approximately one third the population of Michigan (*22*).

### Sample Processing 

We concentrated composite samples of raw wastewater by using filtration, then extracted RNA from the filters (Appendix). We used quantitative reverse transcription PCR (qRT-PCR) to measure the concentration of IAV in wastewater samples (Appendix). The assay targeted RNA coding for the matrix protein 1 (M1) of IAV by using primers and probes developed by the Centers for Disease Control and Prevention (*23*). We used a synthetic influenza H3N2 RNA (Twist Bioscience, https://www.twistbioscience.com) as a standard for comparison. We conducted qRT-PCR to measure the levels of pepper mild mottle virus (PMMoV) within the wastewater (Appendix); PMMoV can indicate the presence of human fecal matter and is used to account for variability in wastewater flow or other physicochemical parameters influencing viral RNA concentration (*24*). We sequenced select IAV amplicons produced through qRT-PCR of RNA extracted from wastewater to validate the identity of the target (Appendix).

### Clinical Data

We obtained influenza hospitalization data for Windsor-Essex through collaboration with the Windsor-Essex County health unit. Using IntelliHealth (https://intellihealth.moh.gov.on.ca) on April 17, 2024, we extracted data from the discharge abstract database and included hospitalization data from September 2022–March 2023. Influenza hospitalizations included hospital admissions where the main diagnoses had a code of J09, J100, J101, J108, J110, J111, or J118 from the International Classification of Diseases, 10th Revision. Windsor-Essex hospitalization data captured all local hospitalizations and included hospitalizations among all residents of Windsor-Essex County, regardless of where the hospitalization occurred. We aggregated influenza hospitalizations by epidemiologic week of initial admission and used hospitalizations per 100,000 persons in subsequent analysis. 

We collected influenza-related hospitalization data for Michigan from the Centers for Disease Control and Prevention’s influenza hospitalization surveillance network (FluSurv-NET), which records laboratory-confirmed influenza-associated hospitalizations during the influenza season as cases per 100,000 persons. We defined influenza-related hospitalization rates as the number of hospitalized persons who tested positive for influenza, of any subtype, through laboratory testing within the 14 days before or during hospitalization (*25*). Hospitalization data were available beginning in October 2022. Although hospitalization data were only available for Clinton, Eaton, Genesee, Ingham, and Washtenaw counties, those data are considered a statewide assessment of influenza for Michigan. Because the WRRF serves approximately one third of the state population, influenza-related hospitalization trends are likely to be reflected in IAV RNA concentrations at the WRRF.

### Data Analysis and Visualization

We used R version 4.3.2 (The R Foundation for Statistical Computing, https://www.r-project.org) for data analysis, including the calculations of Kendall rank correlation coefficient (τ), Spearman rank correlation coefficient (ρ), nonparametric measures of correlation, and nonparametric time lagged cross correlation (TLCC) by using the ccf_boot function in the R package funtimes (Functions for Time Series Analysis, https://cran.r-project.org/web/packages/funtimes). We used Veusz version 3.6.2 (https://veusz.github.io) for data visualization. By using a population-weighted mean, we combined IAV and PMMoV RNA concentration measurements from Windsor-Essex wastewater treatment facilities. We then used downsampling through blockwise averaging to condense the data into a single measurement for each epidemiologic week; this process produced equally spaced data and enabled comparison with hospitalization data available in weekly reports (*26*). Blockwise averaging was not possible for IAV and PMMoV RNA concentration measurements of samples collected from the GLWA-WRRF because samples were collected weekly. No samples were collected from GLWA-WRRF interceptors during epidemiologic week 40 (October 2–8, 2022) and 48 (November 27–December 3, 2022). We filled in the data from epidemiologic weeks 40 and 48 by using linear interpolation before analysis. We used a population-weighted mean to combine the IAV and PMMoV signal for the 3 interceptors that discharge to the GLWA-WRRF, which produced 31 measurements of IAV RNA and 31 measurements of PMMoV RNA concentration for Detroit. All gene concentrations are reported as gene copies (gc) per liter. We produced normalized values for the IAV signal by taking the ratio of IAV M1 gene concentration to the concentration of PMMoV.

We used TLCC with Spearman rank correlations to determine if IAV wastewater signals were leading or lagging indicators of influenza-associated hospitalizations in Windsor-Essex and Michigan. TLCC relies on correlations between data series shifted relative to each other in time and can identify peak synchrony. We determined peak synchrony by the shift that produced the highest Spearman rank correlation coefficient between the 2 timeseries. We also used TLCC to compare the IAV wastewater signal in Windsor-Essex to the IAV wastewater signal in Detroit. We verified the nonnormal data by examining the quantile-quantile plots. We used nonparametric means of correlation, including Kendall rank correlation coefficient and Spearman rank correlation coefficient, to quantify the association between the IAV signal in the wastewater and influenza hospitalizations in both Windsor and Detroit. Correlations between influenza-related hospitalizations and wastewater signal in Detroit were based on 26 weeks of data because hospitalization data were not available until October 2022.

## Results

### IAV M1 Gene Concentrations in Windsor-Essex

Trends in IAV M1 gene concentrations at the monitored plants visually matched trends in influenza-associated hospitalizations in Windsor-Essex for September 2022–March 2023 (Figure). The TLCC for Windsor-Essex showed that wastewater signal lagged new hospital admissions on an epidemiologic week basis (Table 1) and therefore did not provide lead-time. We observed a strong positive relationship between influenza-associated hospitalizations and the population-weighted mean IAV M1 gene concentration (τ = 0.650, p<0.001; ρ = 0.785, p<0.001) (Table 2). Signal normalization with PMMoV RNA concentrations did not improve the association between wastewater signal and influenza-associated hospitalizations (τ = 0.754, p<0.001; ρ = 0.630, p<0.001) or change peak synchrony (Tables 1, 2).

**Figure:**
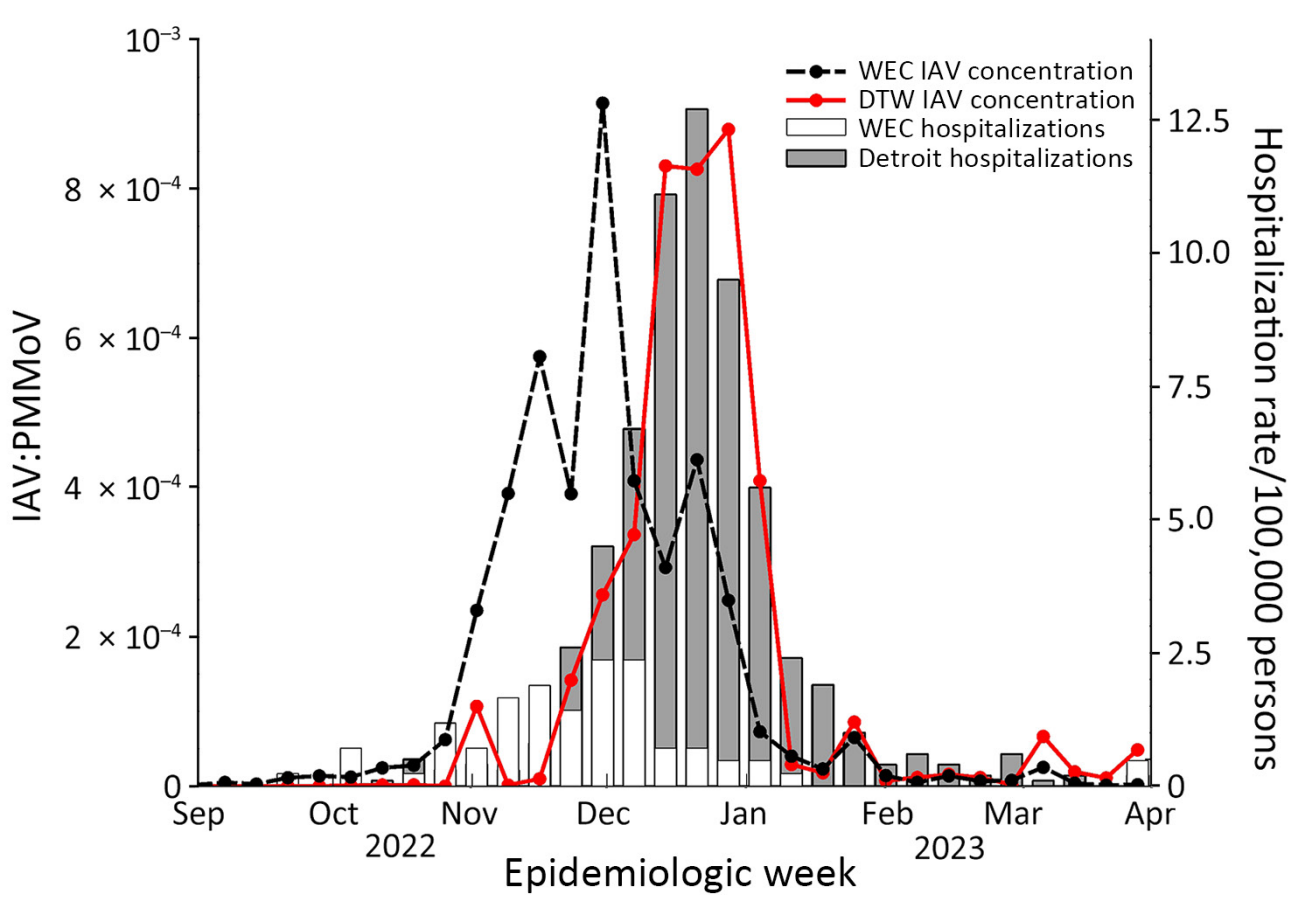
Influenza-associated hospitalization rates and aggregate population-weighted wastewater concentrations for influenza A virus, by epidemiologic week, in Windsor-Essex, Ontario, Canada, and Detroit, Michigan, USA, September 2022–March 2023. The population-weighted PMMoV normalized IAV concentration (lines) is superimposed over the rate of influenza-related hospitalizations (bars). DTW, Detroit wastewater; IAV, influenza A virus; PMMoV, pepper mild mottle virus; WEC, Windsor-Essex County wastewater.

**Table 1:** Temporal shift at which peak synchrony was found between concentrations of influenza A in wastewater and influenza-associated hospitalizations for Windsor-Essex, Ontario, Canada (September 2022–March 2023), and Detroit, Michigan, USA (October 2022–March 2023)*

Associations†	Peak synchrony, wk	Spearman ρ
WEC M1:PMMoV and influenza-associated hospitalizations	1	0.797
WEC M1 and influenza-associated hospitalization	1	0.841
DTW M1:PMMoV and influenza-associated hospitalizations	0	0.708
DTW M1 and influenza-associated hospitalizations	0	0.769
WEC M1:PMMoV and DTW M1:PMMoV	−3	0.642
WEC M1 and DTW M1	−3	0.695

*Peak synchrony was also found between Windsor-Essex and Detroit influenza A wastewater signals during September 2022–March 2023. Wastewater signals were shifted, and clinical metrics remained stationary. Positive values indicate a lagging wastewater signal and negative values indicate a leading wastewater signal. DTW, Detroit wastewater; M1, matrix 1 gene: PMMoV, pepper mottle mild virus; WEC, Windor-Essex County wastewater. †DTW M1, aggregate concentration of influenza A M1 gene (genome copies/mL) in DTW; DTW M1:PMMoV, concentration of influenza A M1 gene in DTW normalized to PMMoV (unitless); WEC M1, aggregate concentration of influenza A M1 gene in WEC (gc/L); WEC M1:PMMoV, concentration of influenza A M1 gene in WEC normalized to PMMoV concentration (unitless).

**Table 2:** Unshifted correlations between influenza-associated hospitalizations and the aggregate population-weighted wastewater concentrations for influenza A virus in Windsor-Essex, Ontario, Canada, September 2022–March 2023*

Associations†	Statistical test results		2-tailed 95% CI‡	2-tailed p value
Kendall τ	Spearman ρ
WEC M1 and influenza-associated hospitalization	0.650		0.482–0.772	<0.001
WEC M1:PMMoV and influenza-associated hospitalizations	0.630		0.456–0.758	<0.001
WEC M1 and influenza-associated hospitalization		0.785	0.589–0. 893	<0.001
WEC M1:PMMoV and influenza-associated hospitalizations		0.754	0.538–0.877	<0.001

*M1, matrix 1 gene: PMMoV, pepper mottle mild virus; WEC, Windor-Essex County wastewater. †WEC M1, aggregate concentration of influenza A M1 gene in WEC (gc/L); WEC M1:PMMoV, concentration of influenza A M1 gene in WEC normalized to PMMoV concentration (unitless). ‡Estimation is based on Fisher r-to-z transformation; estimation of SE is based on the formula proposed by Fieller, Hartley, and Pearson (*27*).

### IAV M1 Gene Concentrations in Detroit

IAV M1 gene concentrations for metro Detroit closely matched the number of new influenza-related hospitalizations in Michigan from October 2022–March 2023 (Figure). We observed a strong positive relationship between influenza-related hospitalizations and the population-weighted mean IAV M1 gene concentration for Detroit (τ = 0.616, p<0.001; ρ = 0.769, p<0.001) (Table 3). The nonparametric TLCC results showed the IAV wastewater signal from Detroit neither lagged nor led influenza-related hospitalizations in the state of Michigan, suggesting the IAV wastewater signal is concordant with influenza-related hospitalizations (Table 1; Figure). Normalization of IAV M1 gene concentrations with PMMoV RNA concentrations did not change the degree of association between the wastewater signal and influenza-related hospitalizations for Michigan (τ = 0.559, p<0.001; ρ = 0.708, p<0.001) or change peak synchrony (Tables 1, 3).

**Table 3:** Unshifted correlations between influenza-associated hospitalizations in Michigan, USA, and the aggregate population-weighted wastewater concentrations for influenza A virus in Detroit, MI, USA from October 2022–March 23*

Associations†	Statistical test results		2-tailed 95% CI‡		2-tailed p value
Kendall τ	Spearman ρ
DTW M1 and influenza-associated hospitalizations	0.616		0.415	0.759	<0.001
DTW M1:PMMoV and influenza-associated hospitalizations	0.559		0.341	0720	<0.001
DTW M1 and influenza-associated hospitalizations		0.769	0.535	0.893	<0.001
DTW M1:PMMoV and influenza-associated hospitalizations		0.708	0. 433	0.863	<0.001

*DTW, Detroit wastewater; M1, matrix 1 gene: PMMoV, pepper mottle mild virus. †DTW M1, aggregate concentration of influenza A M1 gene (genome copies/mL) in DTW; DTW M1:PMMoV, concentration of influenza A M1 gene in DTW normalized to PMMoV (unitless). ‡Estimation is based on Fisher r-to-z transformation; estimation of SE is based on the formula proposed by Fieller, Hartley, and Pearson (*27*).

### Cross-Border Comparison of WS for IAV

The onset and peak of the 2022–23 IAV wastewater signal in Windsor-Essex was observed before the onset and peak of the IAV wastewater signal in Detroit (Figure). TLCC using Spearman rank correlations between the population-weighted weekly average of M1 gene concentrations in Detroit and Windsor-Essex showed the 2022–23 IAV wastewater signal in Detroit lagged the corresponding IAV wastewater signal in Windsor by ≈3 weeks (Table 1). Further comparison by using PMMoV-normalized, population-weighted, weekly averages of the wastewater signal corroborated the lag of ≈3 weeks between Windsor-Essex and Detroit (Table 1).

## Discussion

Our study builds on a growing body of evidence that WS for IAV is highly concordant with the results of other disease incidence measures (*13*,*28*). During the 31-week period of retrospective analysis, influenza-related hospitalizations within Windsor-Essex and Michigan covaried with the concentration of IAV RNA measured in wastewater. However, the IAV signal was not a leading indicator of influenza incidence in either community when analyzed on an epidemiologic week basis; wastewater signal either lagged or was synchronous with hospitalization data. Observation of synchronous or delayed wastewater signal is not without precedent. Recent surveillance efforts have noted lagging wastewater signals (*29*). Other studies have cited the predictive ability of WS in the context of influenza monitoring (*13*,*14*,*30*). In our study, application of blockwise averaging to produce average concentrations of IAV RNA by epidemiologic week could have masked lead time within the data. 

Viral load in influenza patients may peak 1–2 days after symptom onset on the basis of nose and throat swab testing results, and shedding may last 6–7 days (*31*). A meta-analysis of challenge studies examining respiratory tract shedding found shedding lasts an average of 4.8 days, and peak shedding rates occur 2 days after exposure (*32*). A clinical study reported that 41% of IAV-positive patients produced detectable levels of IAV RNA in their feces (*11*). Another study of hospitalized patients found that 47% of people infected with IAV shed IAV RNA in their feces (*33*). Because only some people shed IAV RNA in feces, incubation periods are short, and viral loads rapidly peak, WS loses its ability to predict influenza-associated hospitalizations when the temporal granularity of incidence data is limited. However, producing meaningful data through clinical testing takes longer than results from WS. Case data obtained through laboratory-based virology are released weeks after testing occurs and are often subject to revision because results may reflect data compiled from multiple laboratories. WS can provide more timely measures of incidence because sample processing, RNA quantification, data analysis, and reporting are often completed the same day as sample collection in Windsor-Essex. WS can be considered de facto lead-time because data may be disseminated to public health officials well in advance of case data.

The utility of WS is not restricted to predictive ability. WS is an independent and sensitive measure of disease prevalence (*34*), enabling it to be used as an additional metric for trend comparison across jurisdictional boundaries, and it may be helpful when testing conventions and public health policies differ. Unlike WS, influenza cases and hospitalizations likely represent only the most severe cases of influenza in which people sought medical testing and treatment, and they do not necessarily represent population-wide trends. WS has the potential to aid in accurately tracking infection dynamics when testing capacity is limited or few patients seek medical care. 

In the case of Windsor-Essex and Detroit, the cross-border movement of persons and goods is vital to the region because of strong economy integration (*35*). Many people, such as healthcare and automotive workers, commute across the border daily to work in Michigan while living in Ontario (*16*). Almost 18,000 people crossed into Windsor-Essex from Detroit daily over the course of our study, which suggests the communities could have concurrent influenza seasons (Appendix Figure 1). Contrary to this expectation, we observed the peak and onset of the 2022–23 IAV wastewater signal in Detroit was delayed by ≈3 weeks when compared with Windsor-Essex. An explanation for this discrepancy could be the lingering effect of travel restrictions implemented during the COVID-19 pandemic, which limited travel between these interconnected cities (Appendix Figure 1). There was no restriction on trade or the commutes of essential workers, and testing requirements for cross-border travel ended in April 2022. The remaining COVID-19 related travel restrictions were lifted at the start of October 2022, before the onset of the influenza season. Despite the removal of restrictions, the number of people crossing into Windsor-Essex each day during the second half of 2022 was ≈25% less than the number of people crossing prior to the COVID-19 pandemic (17,867 vs. 24,260) (Appendix Figure 2). The residual effect of border restrictions, evidenced by the suppressed cross-border traffic in the leadup to the 2022–23 respiratory season, shows that border restrictions could have played a role in the observed discrepancies between Windsor-Essex and Detroit.

Another potential cause of the timing difference in the influenza seasons between Detroit and Windsor-Essex could be pandemic mitigation strategies, such as mask mandates and social distancing guidance. Ontario was slower to implement a mask mandate but kept the mandate in place much longer than Michigan did. Michigan lifted all masking requirements on June 22, 2021, whereas Ontario ended its mask mandate 272 days later, on March 21, 2022 (*36*,*37*). Michigan ending the mask mandate early could have enabled the circulation of influenza in Detroit before the 2022–23 respiratory season, increasing levels of natural immunity in Michigan. However, clinical data and wastewater surveillance in Windsor-Essex show that influenza was circulating in Windsor-Essex in spring 2022 (Appendix Figure 3). 

Differences in influenza immunization campaigns might also explain the differences in influenza season onset between Detroit and Windsor-Essex. Influenza vaccines could have played a role in determining the effective reproduction number for IAV in the 2022–23 season. Preliminary research showed the vaccine effectiveness (VE) in Canada against IAV subtype H3N2 was 54% for people <65 years of age (*17*). H3N2 was the dominant IAV subtype, representing ≈95% of cases (*17*), in contrast with the limited sequencing results of a selection of amplicons (Appendix Figure 4). Similar estimates of VE for this cohort were produced for Wisconsin (60%) (*38*) and across the United States (51%) (*39*). Michigan and Ontario use similar vaccines, with a focus on administering quadrivalent-inactivated influenza vaccines to the population (*40*,*41*). Although inoculation with some vaccine types, such as live attenuated vaccines, is associated with viral shedding, it is unlikely to contribute to wastewater signals (*42*). Because VE and vaccine type were similar between Michigan and Ontario, the differentiating factor could be inoculation timing. Influenza vaccine distribution in Michigan typically begins earlier than Ontario, with inoculations happening as early as July. By November 2022, a total of 2,632,430 Michigan residents were vaccinated (≈25% of the population) (*43*). Inoculation efforts in Ontario began later, and vaccines were not made available to all residents until November (*44*). Mass influenza inoculation efforts in Ontario began after IAV RNA concentration started increasing in the wastewater, signaling the start of the influenza season. Vaccination campaigns were already well under way in Michigan when the IAV RNA concentration began to increase in wastewater. We speculate that people from Ontario were less likely to have vaccine-induced immunity than those from Michigan early in the season, potentially contributing to the earlier peak in Windsor-Essex wastewater IAV signal. However, it is unclear if the timing of vaccine campaigns contributed to the observed difference between the Michigan and Ontario influenza seasons. Additional factors, including socio-economic status (*45*), access to healthcare, racial demographics (*46*), population age structure (*47*), and virus–virus interactions (*48*) could have contributed to the differences. Population-level administration schedules for seasonal influenza vaccines merit further investigation; those schedules help determine when herd immunity is reached and if herd immunity is reached before the spread of illness within a community. 

The first limitation of our study is that WRRF does not serve the Michigan counties where FluSurv-NET–participating hospitals are located. However, both the hospitalization data garnered from FluSurv-Net and wastewater data can be considered a proxy for statewide incidence. All influenza-associated hospitalizations were included in this dataset, for both Michigan and Windsor-Essex, even though the WS focused only on IAV. Our analysis could have been affected by focus on IAV, despite its dominance in the 2022–23 influenza season (*17*). Not all hospitalizations recorded in the Windsor-Essex data were laboratory confirmed cases of influenza. The temporal resolution of sample collection at the WRRF was limited, and weekly sampling might have failed to capture variability in the wastewater signal. Finally, the wastewater treatment plants monitored in both Windsor-Essex and Detroit, although serving representative populations, do not encompass all the residents and could have failed to capture variability in IAV wastewater signal.

In conclusion, our study demonstrates how wastewater surveillance can shed light on regional differences that may have otherwise gone unnoticed, or remain unvalidated, because of the inherent limitations of traditional metrics to capture populationwide trends. Future studies investigating influenza vaccine administration schedules should incorporate WS as an independent metric of disease incidence. WS can potentially provide more timely measures of incidence, rather than waiting for the release of laboratory testing. The utility of WS as a predictive metric, and as a metric for trend comparison across jurisdictional boundaries with different approaches to vaccination and collecting disease incidence metrics, demonstrates its usefulness when testing conventions and public health policies differ.

Appendix Additional information about wastewater surveillance confirms differences in influenza A infection between Michigan, USA, and Ontario, Canada, September 2022–March 2023.
