# Supplementary material for: Wastewater Surveillance to Confirm Differences in Influenza A Infection between Michigan, USA, and Ontario, Canada, September 2022–March 2023
Source: Emerg Infect Dis. 2024 Aug;30(8):1580–8. doi: 10.3201/eid3008.240225 (PMC11286066; doi:10.3201/eid3008.240225)
Supplement: Appendix — Additional information about wastewater surveillance confirms differences in influenza A infection between Michigan, USA, and Ontario, Canada, September 2022–March 2023. [file 24-0225-Techapp-s1.pdf]

Article DOI: https://doi.org/10.3201/eid3008.240225

EID cannot ensure accessibility for supplementary materials supplied by authors. Readers who have difficulty accessing supplementary content should contact the authors for assistance.

Wastewater Surveillance to Confirm Differences in Influenza A Infection between Michigan, USA, and Ontario, Canada, September 2022–March 2023

Appendix

Appendix Methods

Sample Collection

Untreated wastewater samples were collected five times per week from two different wastewater treatment plants located in Windsor-Essex between September 1st, 2022, and March 31, 2023. Sample collection spanned a 31-week period. The Lou Romano Water Reclamation Plant (LRWRP) serves ≈180,000 individuals and is the largest treatment facility in Windsor-Essex. The Little River Pollution Control Plant (LRPCP) serves 90,000 persons and together with LRWRP these plants service the majority of Windsor-Essex's urban center. Concurrently, samples were collected from the Water Resource Recovery Facility (WRRF) operated by the Great Lakes Water Authority (GLWA) located in Detroit, MI at a frequency of one sample per week. The WRRF facility serves the majority of the greater metropolitan Detroit area and treats the waste of ≈3 million individuals, which equates to roughly a third of the population of the state of Michigan. The plant treats the combined stormwater, industrial, residential, and commercial waste that arrive through three major interceptors. These are the Detroit River Interceptor, the North Interceptor-East Arm and the Oakwood-Northwest-Wayne County Interceptor which serve a large region of southeast Michigan including the City of Detroit. All samples collected in both Windsor-Essex and Detroit were 1L 24-hour composite samples composed of aliquots of wastewater removed from the influent stream regular intervals. Following collection, samples were transported on ice to the laboratory for immediate concentration and analysis.

Sample Processing

Composite samples of raw wastewater were concentrated by filtering 50–120 mL through 0.22 µm Sterivex PES cartridge filters (MilliporeSigma, Burlington, MA, USA) using a 60 mL syringe fitted into a caulking gun. Immediately following filtration, the filters were sealed and flash-frozen through immersion in liquid nitrogen. Subsequently, filters were subjected to downstream processes including RNA extraction and RT-qPCR.

Following filtration and flash freezing, filters were thawed, and the filter membrane was cut from the Sterivex cartridge using a sterile scalpel and forceps. Total nucleic acid was extracted from the filter membranes using either the AllPrep PowerViral DNA/RNA kit (Qiagen, Germantown, MD, USA) modified by addition of 5% 2-mercaptoethanol (v/v) or the RNeasy PowerMicrobiome Kit (Qiagen, Germantown, MD, USA), again modified by addition of 5% 2-mercaptoethanol (v/v). Samples were not treated with DNase upon extraction and RNA was eluted in 50µL of RNase free water.

RT-qPCR

An RT-qPCR assay was used to measure the concentration of influenza A virus (IAV) in wastewater samples. The assay targeted RNA that codes for the matrix protein 1 (M1-gene) of IAV using primers and probes developed by the U.S. CDC (*1*). Primers and probes were supplied by Integrated DNA Technologies (Coralville, IA, USA) and primer and probe sequences can be found in Supplementary Table 1.

Appendix Table 1. Primer/probe sequences for RT-qPCR of IAV M1 gene

IAV Assay	Sequence
Forward Primer	5′-CAAGACCAATCYTGTCACCTCTGAC-3′
Reverse Primer	5′-GCATTYTGGACAAAVCGTCTACG-3′
Probe	5′-/FAM/TGCAGTCCT/ZEN/CGCTCACTGGGCACG/3IABkFQ/-3′

Reactions contained 4µL of RNA template mixed with 10µL of Luna Universal Probe One-Step Reaction Mix (2X), 1µL Luna WarmStart® RT Enzyme Mix (20X) (Luna® One-Step RT-qPCR Kit, Massachusetts, USA), forward primer (final concentration of 500nM), reverse primer (final concentration of 500nM), and probe (final concentration of 250nM) in a final reaction volume of 20µL. RT was performed at 55°C for 10 min, followed by polymerase activation at 95°C for 1 min, and 45 cycles of denaturation, annealing/extension at 95°C for 10 sec, then 55°C for 45 sec, respectively. No template controls yielded no amplification, and the limit of detection for the assay was determined at 4 gene copies of IAV per reaction containing

4μL of template RNA, corresponding to a greater than 95% probability of detection. LOD was determined through analysis of 20 replicate 8-point standard curves. Twist Synthetic Influenza H3N2 RNA control (Twist Bioscience, San Francisco, CA) was used to create an 8-point standard curve to quantify gene targets. RT-qPCR assays were also performed to evaluate the levels of Pepper Mild Mottled Virus (PMMoV) within the wastewater. PMMoV is a widely accepted indicator of the presence of human fecal matter (*2–4*). For quantification of PMMoV, reactions contained 2.5μL of RNA template mixed with 10μL of Luna Universal Probe One-Step Reaction Mix (2X), 1μL Luna WarmStart® RT Enzyme Mix (20X) (Luna® One-Step RT-qPCR Kit, Massachusetts, USA), 3.5μL of water and the remaining 3μL consisted of forward primer, reverse primer, and probe each with a final concentration of 200nM. Primers and probes for the amplification of PMMoV were previously described (*5*). Reverse transcription was performed for 10 minutes at 55°C, this was followed by an enzyme activation step at 95°C for 1 minute and 40 cycles of denaturation and annealing/extension at 95°C for 10 seconds and 55°C for 30 seconds respectively. No template controls were included with each plate of RT-qPCR run and whole process controls were included with each extraction. The 7-point standard curve for the quantification of PMMoV was generated through serial dilution of a custom gBlock (Integrated DNA Technologies, Coralville, IA, USA) and was run with each plate of samples. No amplification was observed either process controls (extraction blanks) or in no template controls. Reaction inhibition was assessed using VetMAX XENO Internal Positive Control RNA (Applied Biosystems Corp., Waltham, MA, USA). VetMax template was spiked into water (which was used as a reference), undiluted DNA/RNA extracts, DNA/RNA extracts diluted 1:5, and DNA/RNA extracts diluted 1:10. Recovery was compared between conditions, and it was determined that inhibition could be addressed through dilution. Due to repeated incidence of inhibition with wastewater samples processed by filtration, template was diluted 1:5 or 1:10 in all reactions. Technical triplicates were run for detection of gene targets. Thermal cycling was performed using a MA6000 qPCR thermocycler (Sansure Biotech, Changsha, China).

Amplicon Sequencing

To validate the identity of the amplicon obtained from M1 gene of IAV, RTq-PCR products obtained from IAV-positive wastewater samples were sequenced. First, the PCR products were cleaned up by adding 1 volume of NEBNext® Sample Purification Beads (NEB).

DNA was eluted in 15 µL Nuclease-free water and quantified using Denovix DS-11 Spectrophotometer.

Second, the end-prep reaction was performed employing 250–300 ng of DNA mixed with 1.75 µL UltraII End Prep Buffer, 0.75 µL UltraII End Prep Enzyme and water to a final volume of 15 µL. The reaction was incubated at 20°C for 10 minutes and 65°C for 10 minutes, holding at 4°C. Barcoding of samples was carried out combining 3 µL of end-prepped sample, 2.5 µL Native Barcode (ONT, native barcoding EXP-NBD104), 10 µL Blunt/TA Ligase Master Mix and 4.5 µL nuclease-free water. Samples were pooled after barcoding and then analyzed in a single sequencing run with separated reads obtained from each of the amplicons. Ligation reaction was incubated at 22°C for 20 minutes and 65°C for 10 minutes, followed by a hold on ice for at least 1 minute. Cleaning up of the ligated sample was performed by adding 0.4 volume NEBNext® Sample Purification Beads and eluting with 12 µL of nuclease-free water. The Oxford Nanopore sequencing adaptor ligation was performed employing 200 ng of barcoded DNA in 30 µL, mixed with 5 µL Adaptor Mix II, 10 µL 5X NEBNext Quick Ligation Reaction Buffer (NEB) and 5 µL Quick T4 DNA Ligase (NEB). The incubation was carried out at 25°C for 30 minutes. Sample was cleaned up by adding 1 volume of NEBNext® Sample Purification Beads, eluted in 12 µL of Elution Buffer and quantified. Twenty ng of the library was loaded onto a SpotOn Flow Cell (R9.4 flow cell). Data was collected along 16 hours of sequencing with MinION.The FastQ files containing the sequenced reads were uploaded in Epi2me desktop application (Oxford Nanopore Technologies). Reads were analyzed employing the WIMP (*What's In My Pot*) workflow (v2023.06.13–1865548) setting filters for reads length between 100 bp to 250 bp. WIMP initially filters FASTQ files with a mean q-score below a minimum threshold (defaults to 7). For reads above the quality threshold, the Centrifuge classification engine is executed to assign each read to a taxon in the NCBI taxonomy. Taxonomical assignment is done based on the scores calculated by the microbial classification engine called Centrifuge using default settings where the minimum length of partial hits is set to 25 (min_hit_len) and the minimum summed length of partial hits is set to 0 (*6*). Manual calculation of the identity % for the taxonomical assignments was not completed. The Centrifuge classification results are then filtered and aggregated to calculate and report counts of reads at the species rank. For reads without a reliable assignment at the species rank, higher ranks of the

taxonomy tree are used for the assignment. Supplementary Figure 4 shows a taxonomic tree of IAV sequences detected through Nanopore sequencing of the amplicons.

Additionally, several filtered sequences were randomly selected and manually uploaded to Basic Local Alignment Search Tool (BLAST) to confirm the identity of the M1 gene portion amplified by RT-qPCR. Raw sequencing data was uploaded to the National Center for Biotechnology Information (NCBI) Sequence Read Archive (SRA). Raw sequence data have the following accessions: SAMN39936024, SAMN39936025, SAMN39936026, SAMN39936027, and SAMN39936028.

References

1. Centers for Disease Control and Prevention. CDC's influenza SARS-CoV-2 multiplex assay. 2020 [cited 2023 Sep 20]. https://www.cdc.gov/coronavirus/2019-ncov/lab/multiplex.html

2. Rosario K, Symonds EM, Sinigalliano C, Stewart J, Breitbart M. Pepper mild mottle virus as an indicator of fecal pollution. Appl Environ Microbiol. 2009;75:7261–7. PubMed https://doi.org/10.1128/AEM.00410-09

3. Kitajima M, Sassi HP, Torrey JR. Pepper mild mottle virus as a water quality indicator. NPJ Clean Water. 2018;1:1–9. https://doi.org/10.1038/s41545-018-0019-5

4. Zhang T, Breitbart M, Lee WH, Run JQ, Wei CL, Soh SWL, et al. RNA viral community in human feces: prevalence of plant pathogenic viruses. PLoS Biol. 2006;4:e3. PubMed https://doi.org/10.1371/journal.pbio.0040003

5. Haramoto E, Kitajima M, Kishida N, Konno Y, Katayama H, Asami M, et al. Occurrence of pepper mild mottle virus in drinking water sources in Japan. Appl Environ Microbiol. 2013;79:7413–8. PubMed https://doi.org/10.1128/AEM.02354-13

6. Kim D, Song L, Breitwieser FP, Salzberg SL. Centrifuge: rapid and sensitive classification of metagenomic sequences. Genome Res. 2016;26:1721–9. PubMed https://doi.org/10.1101/gr.210641.116

7. Canada Border Services Agency. Traveller volumes by port of entry and month. 2024 [cited 2024 Jan 30]. https://open.canada.ca/data/dataset/1b1c2b92-b388-47d9-87d4-01aee8d3c3e4

8. The Windsor-Essex County Health Unit. Weekly Influenza Bulletin Data Dashboard. [cited 2023 Sep 28]. https://www.wechu.org/reports/weekly-influenza-bulletin

9. Skowronski DM, Chuang ES, Sabaiduc S, Kaweski SE, Kim S, Dickinson JA, et al. Vaccine effectiveness estimates from an early-season influenza A(H3N2) epidemic, including unique genetic diversity with reassortment, Canada, 2022/23. Euro Surveill. 2023;28:2300043. PubMed https://doi.org/10.2807/1560-7917.ES.2023.28.5.2300043

10. Mercier E, D'Aoust PM, Thakali O, Hegazy N, Jia JJ, Zhang Z, et al. Municipal and neighbourhood level wastewater surveillance and subtyping of an influenza virus outbreak. Sci Rep. 2022;12:15777. PubMed https://doi.org/10.1038/s41598-022-20076-z

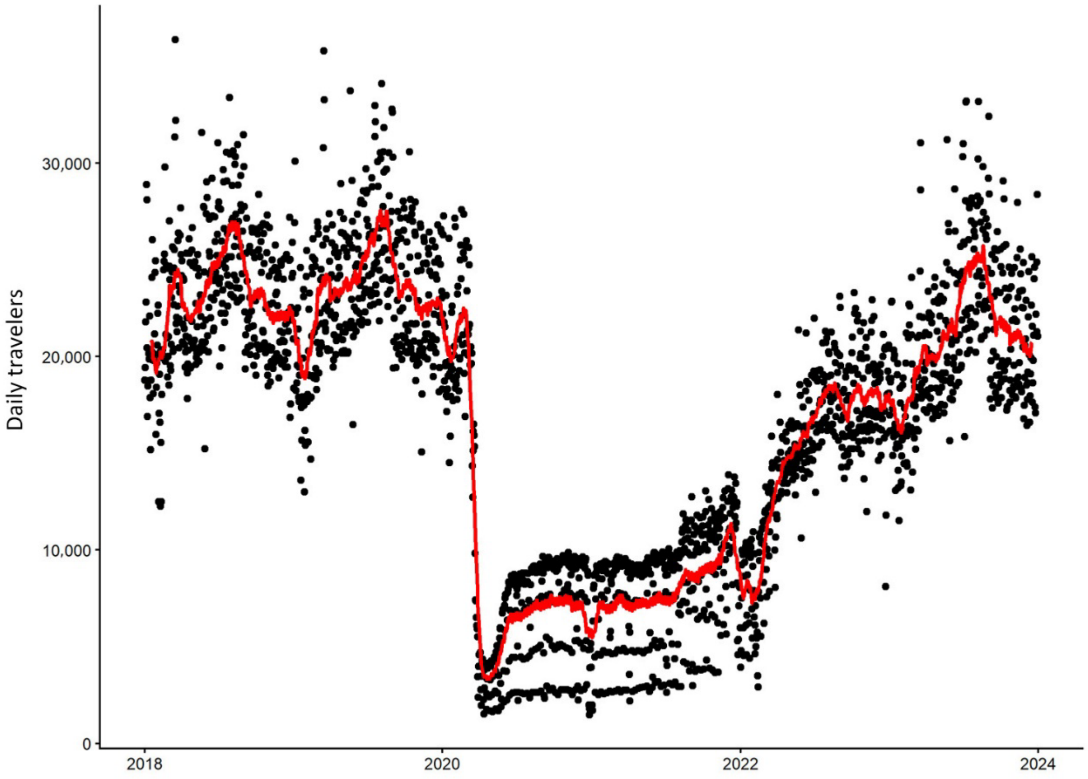

Appendix Figure 1. Daily count of travellers crossing into Windsor-Essex, ON Canada from Detroit at land crossings from January 1, 2018, to December 30, 2023. Dots correspond to the sum of traffic arriving through the Detroit-Windsor tunnel and the Ambassador Bridge. The red line is a 30-day moving average of the daily arrivals. The onset of the COVID-19 pandemic coincides with a precipitous decline in the number of travellers arriving in Windsor-Essex daily. Traveller volume data was publicly available (*7*).

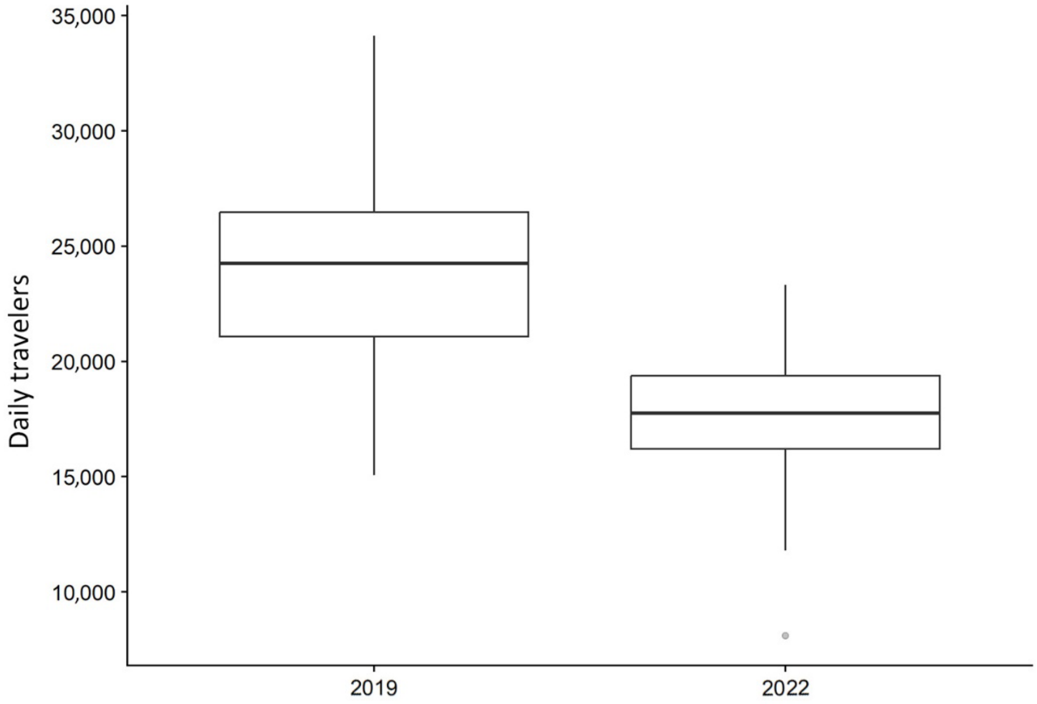

Appendix Figure 2. Comparison of the mean number of daily travellers arriving in Windsor-Essex at land crossings (Detroit-Windsor tunnel and the Ambassador Bridge) from August to December of 2019 (before the COVID-19 pandemic) to the mean number of daily travellers arriving in Windsor-Essex at land crossings from August to December of 2022 (following the removal of COVID-19 pandemic restrictions). The results of a paired *t*-test show that ≈6394 ($p < 0.0001$, 95% CI 5720–7068) fewer daily travellers crossed into Windsor-Essex in 2022 than in the same period of 2019 (a mean of 24,260 travellers in 2019 compared to 17,867 travellers in 2022). This represents an approximate 25% reduction in cross-border traffic during the onset and peak of the 2022–23 respiratory season despite the removal of border restrictions. Traveller volume data were publicly available (*7*).

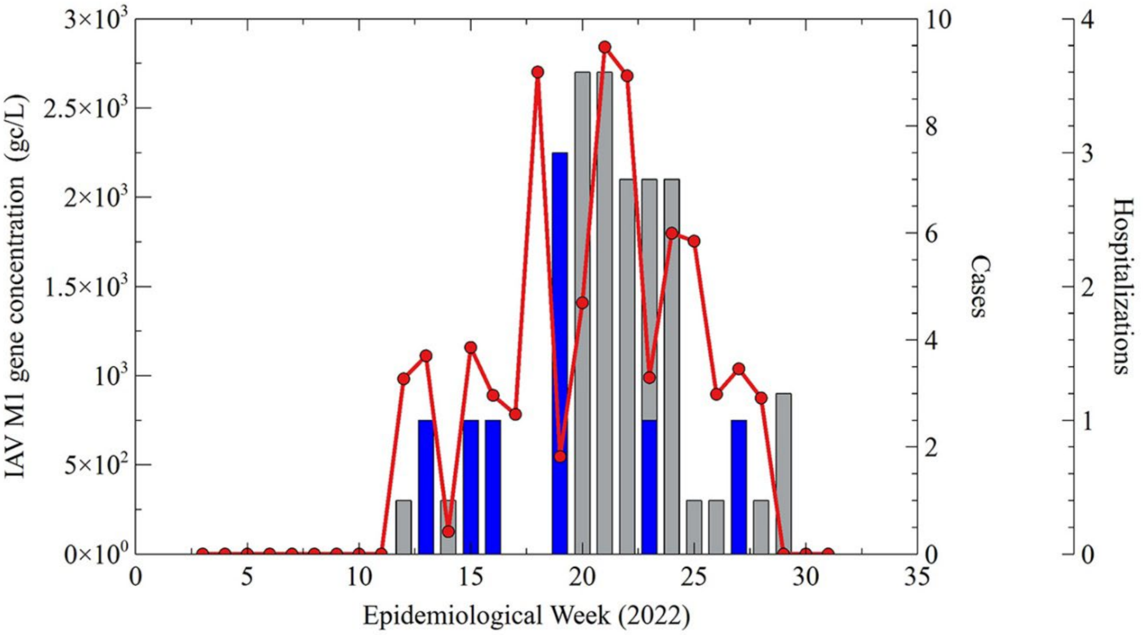

Appendix Figure 3. IAV-M1-gene concentration (red line) superimposed on new influenza hospitalizations for the Windsor-Essex Region (blue bars) and IAV cases (gray bars) per epi-week for 2022. Hospitalization data were extracted from the Discharge Abstract Database (DAD) through IntelliHealth. Influenza hospitalizations included hospital admissions where the main diagnoses had an ICD10 code of J09, J100, J101, J108, J110, J111, or J118. Case data were found on the Windsor-Essex County Health Unit's Public Dashboard (*8*). Unpublished wastewater surveillance data corroborates an abnormal peak in influenza cases and hospitalizations observed in May 2022 following the removal of COVID-19 mitigation measures. This late season spike in influenza circulation was observed across Canada (*9*,*10*).

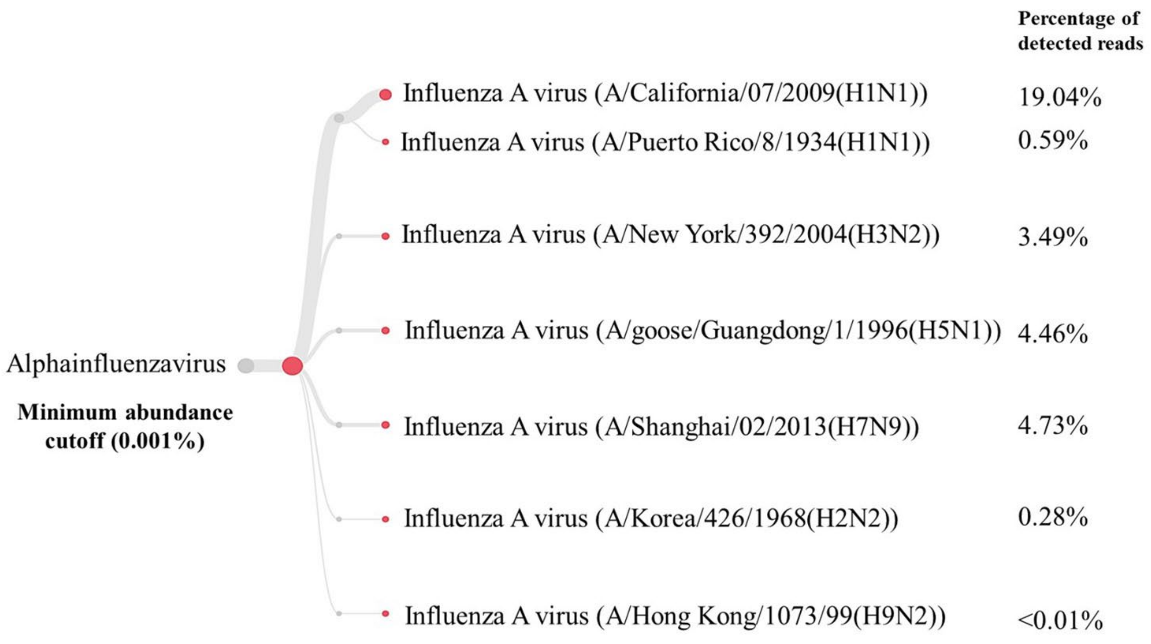

Appendix Figure 4. Taxonomy tree of sequences detected through Nanopore sequencing of the amplicons obtained by RT-qPCR. All the IAV subtypes with at least 0.001% of abundance are included in this taxonomic representation.
